# Association Between Dairy Products Consumption and Esophageal, Stomach, and Pancreatic Cancers in the PANESOES Multi Case–Control Study

**DOI:** 10.3390/cancers16244151

**Published:** 2024-12-12

**Authors:** Alejandro Oncina-Cánovas, Laura Torres-Collado, Manuela García-de-la-Hera, Laura María Compañ-Gabucio, Sandra González-Palacios, Antonio José Signes-Pastor, Jesús Vioque

**Affiliations:** 1 Instituto de Investigación Sanitaria y Biomédica de Alicante, Universidad Miguel Hernández (ISABIAL-UMH), 03010 Alicante, Spain; aoncina@umh.es (A.O.-C.); manoli@umh.es (M.G.-d.-l.-H.); lcompan@umh.es (L.M.C.-G.); sandra.gonzalezp@umh.es (S.G.-P.); asignes@umh.es (A.J.S.-P.); 2 Unidad de Epidemiología de la Nutrición, Departamento de Salud Pública, Historia de la Ciencia y Ginecología, Universidad Miguel Hernández (UMH), 03550 Alicante, Spain; 3 CIBER Epidemiología y Salud Pública (CIBERESP), Instituto de Salud Carlos III, 28034 Madrid, Spain

**Keywords:** dairy products, fermented dairy, sugary dairy desserts, cancer, esophagus, stomach, pancreas

## Abstract

**Background/Objectives:** This study explored the association between dairy products consumption (total and subgroups) and cancer of the esophagus, stomach, and pancreas within the PANESOES case–control study. **Methods:** Data from 1229 participants, including 774 incident cases of cancer and 455 controls matched by age, sex, and region, were analyzed. Dietary intake was assessed using a validated Food Frequency Questionnaire, categorizing dairy intake by total and subgroups (fermented dairy, sugary dairy desserts, and milk). Multinomial logistic regression was used to estimate relative risk ratios (RRRs), adjusting for confounders. **Results:** We found an inverse association between moderate dairy consumption (T2) and esophageal cancer (RRR _T2 vs. T1_ = 0.59 (95%CI: 0.37–0.96)). The highest tertile (T3) of fermented dairy was associated with a lower risk of esophageal (RRR _T3 vs. T1_ = 0.55 (0.33–0.90)) and stomach cancers (RRR _T3 vs. T1_ = 0.68 (0.47–0.97)). By contrast, the highest tertile of consumption of sugary dairy desserts was associated with a higher risk of stomach cancer (RRR _T3 vs. T1_ = 1.85 (1.30–2.64)). No association was found for milk. **Conclusions:** This study suggests that fermented dairy may reduce the risk of esophageal and stomach cancers, while sugary dairy desserts may increase the risk of stomach cancer.

## Simple Summary

Digestive cancers are still important contributors to the global burden of cancer in our society, and diet may play a role in their development. In our study, we observed an association between dairy products, especially fermented ones, and a lower risk of esophageal and stomach cancers, whereas high intake of sugary dairy desserts was linked to a higher risk of stomach cancer. Fermented dairy products, which contain beneficial compounds, such as probiotics, appear to offer a protective effect, particularly at higher levels of consumption. In contrast, high consumption of sugary dairy desserts, commonly rich in free sugars and unhealthy fats, was associated with a higher risk of stomach cancer. No significant association was found with milk consumption. These findings suggest that the risk of upper digestive tract cancers may be mitigated by consuming fermented dairy, while high intake of sugary dairy desserts could elevate this risk.

## 1. Introduction

Cancer remains one of the leading causes of morbidity and mortality worldwide, with nearly 20 million new cases diagnosed in 2022 resulting in around 10 million deaths [1]. In addition to breast cancer in women and lung and prostate cancers in men, cancers of the digestive tract remain a global health problem because of their high mortality rates and insufficiently known etiology. In this sense, esophageal cancer is still a leading cause of cancer deaths globally, with two main histological subtypes, such as esophageal squamous cell carcinoma and esophageal adenocarcinoma. Similarly, stomach cancer is among the most common malignancies worldwide, while pancreatic cancer is characterized by its extremely low survival rate, primarily due to late diagnosis and a lack of effective treatment options [2]. These cancers share some common risk factors, including tobacco and alcohol consumption, as well as certain dietary factors [3]. Thus, dietary factors have been linked to the development of many cancers, including those of the esophagus, stomach, and pancreas [4]. A diet rich in whole grains, fruits, and vegetables has been associated with a lower risk of esophageal and stomach cancers, likely due to their high content of fiber, antioxidants, and anti-inflammatory compounds [5]. In contrast, other factors, such as red and processed meats, have been classified as carcinogenic in colorectal cancer (Group 1) by the International Agency for Research on Cancer (IARC) [6], and they have been linked to an increased risk of esophageal and stomach cancers as well [7,8]. This association may be due to the harmful chemicals produced during meat processing and cooking [9]. The role of other food groups, such as dairy products, is less clear, with inconsistent results observed, and their potential impact on these cancers has yet to be elucidated [10].

Certain components of dairy products may exert a protective effect on the risk of esophageal, stomach, and pancreatic cancers through various mechanisms. Vitamin D, one of the most studied components, may have anticancer effects through its regulation of cell growth, apoptosis, and immune function [11]. Calcium, another important nutrient in dairy products, may reduce cancer risk by inhibiting the proliferation and differentiation of cancer cells [12]. Additionally, the probiotics found in fermented dairy products, such as yoghurt, may modulate gut microbiota, reducing inflammation and enhancing immune responses, which could lower the risk of digestive tract cancers [13]. However, the effects of these components may vary depending on the type of dairy product consumed and individual factors, such as genetics and gut health.

Previous studies have investigated the association between dairy products consumption and the risk of the three digestive cancers of interest, reporting inconsistent results. A recent meta-analysis of 34 prospective studies, involving more than 3 million participants, showed no association between total dairy consumption and cancer mortality [14]. However, higher milk consumption was associated with a 10% higher risk of cancer mortality in women, while fermented milk consumption was associated with a 15% lower risk of cancer mortality in women. In addition, cheese consumption was associated with a 22% higher risk of colorectal cancer mortality [14]. Other studies have also examined the association between the consumption of total or specific dairy products and the risk of esophageal, stomach, and pancreatic cancers, showing inconsistent results. The Japan Collaborative Cohort Study showed no significant link between dairy intake and the risk of pancreatic [15] and esophageal [16] cancers. A pooled analysis from the StoP Consortium found no association between yoghurt intake and gastric cancer risk [17]. Two meta-analyses—one by Li et al. [18] exploring the relationship between dairy consumption and esophageal squamous cell carcinoma risk and the other by Wang et al. [19] examining the association between milk and dairy product consumption and gastric cancer risk—also found no consistent results for these associations. Therefore, further studies are needed to clarify the role of total dairy and specific dairy products in the risk of these cancers. Thus, we aimed to explore the association between dairy products consumption (total and subgroups) and cancers of the esophagus, stomach, and pancreas within the multi case–control PANESOES study.

## 2. Materials and Methods

### 2.1. Study Design and Population

The PANESOES study was a hospital-based multi case–control study aimed at exploring the effect of major lifestyle factors and diet on the risk of three types of digestive cancers: esophageal, stomach, and pancreatic [5,20,21,22]. The participants included women and men from Spain aged between 30 and 80 years, recruited after their hospitalization between January 1995 and March 1999. A total of 9 hospitals participated in the study, all located in the province of Alicante (Hospital General, Hospital Clínico de San Juan, Hospital de Elche, and Hospital Comarcal de la Vega Baja) and Valencia (Hospital Clínico Universitario, Hospital La Fe, Hospital Dr. Peset, and Hospital Arnau de Vilanova).

The cases included patients newly diagnosed with one of the three cancers, confirmed cyto-histologically (esophageal, stomach, and pancreatic) and/or by clinical evidence (pancreatic). A total of 774 cases (199 esophageal, 411 stomach, and 164 pancreatic), with complete information, were included in the present analysis. The controls were frequency-matched to cases by age (three categories: <60; 60–69; and >70 years), sex, and province (Alicante and Valencia). The control group was selected through a broad inclusion criterion, which covered diseases a priori non-related to the main exposures of interest (tobacco, alcohol, and diet). The overall participation rate of the controls was 99.6% (455 controls). The distribution of the main diagnostic groups of control subjects was hernias (35.9%), fractures or injuries (32.4%), appendicitis (6.7%), incisional hernias (2.5%), acute cholecystitis (1.4%), and other diagnoses (21.1%).

All subjects were informed of the study objectives and gave their informed consent before inclusion in the study. The research protocols were approved by the local ethics and/or research committees of the participating hospitals and the university (AUT.DSP.JVL.04.21).

### 2.2. Dietary Intake and Dairy Product

Trained interviewers collected data on habitual dietary intake using a validated semi-quantitative Food Frequency Questionnaire (FFQ) [23] designed in a similar way to the Harvard questionnaire [24]. During the hospital interview, participants were asked how often, on average, they had consumed each food item in the FFQ over a whole year, referring to the 5 years before the hospital interview. The FFQ comprised 93 food items with 9 consumption frequency options, ranging from “Never or less than once a month” to “6 or more times a day”. The dairy section included 9 items with the following serving sizes:

- Whole-fat milk (1 glass or cup, 200 cc);
- Skimmed milk (1 glass or cup, 200 cc);
- Condensed milk (1 tablespoon);
- Yoghurt (One, 125 g);
- Cottage cheese, curd, white or fresh cheese (100 g);
- Creamy cheese or cheese in portions (One portion);
- Mature or semi-mature cheese: Manchego (1 piece, 50 g);
- Custard, flan, pudding (One);
- Ice cream (1 cone, cup, or scoop).

As not all dairy products are similar nutritionally (some are produced through a fermentation process, while others contain added sugar and/or fats) and can thus have different effects [25], consequently, we grouped dairy products into three dairy subgroups: fermented dairy, sugary dairy desserts, and milk. Table 1 summarizes the items included for each dairy subgroup. In the present study, we use both the total dairy group and the three dairy subgroups, measured in grams per day and classified into tertiles of consumption.

### 2.3. Other Variables

Information regarding the participants’ sociodemographic and lifestyle characteristics was also considered: age (<60; 60–69; and >70 years), sex (male or female), province (Alicante or Valencia), educational level (<primary; primary; >primary), smoking (never smoked; ex-smoker; ≤24 cigarettes per day; >24 cigarettes per day), alcohol consumption (never; 1–24 g per day; 25–49 g per day; 50–99 g per day; >99 g per day), daily energy intake (in kilocalories), and daily fruit and vegetable consumption (in grams per day).

### 2.4. Statistical Analysis

We used ANOVA (for quantitative variables, including mean and standard deviation [SD]) and chi-square tests (for categorical variables) to compare the characteristics between the included cases and controls. To examine the association between dairy consumption (total and subgroups) and esophageal, stomach, and pancreatic cancers, we used multinomial logistic regression, presenting two models to adjust for potential confounding variables. Model 1 included the matching variables: age (three categories: <60; 60–69; and >70 years), sex (male or female), and province (Alicante and Valencia). Model 2 additionally included variables identified through a comprehensive review of the existing literature: educational level (<primary; primary; >primary), smoking (never smoked; ex-smoker; ≤24 cigarettes per day; >24 cigarettes per day), alcohol consumption (never; 1–24 g per day; 25–49 g per day; 50–99 g per day; >99 g per day), daily energy intake (in kilocalories), and daily fruit and vegetable consumption (in grams per day).

We used the likelihood ratio test to assess the overall significance of both total and subgroups of dairy products consumption and the Wald test for specific categories. We also performed trend tests to explore the dose–response relationship (*p*-trend). All analyses were conducted using STATA software, version 17.

## 3. Results

The main characteristics of cases and controls are presented in Table 2. Esophageal cases were younger and showed the highest consumption of both tobacco and alcohol but the lowest consumption of fruit and vegetables, total dairy, and fermented products. Stomach cases showed the highest consumption of total dairy products, dairy desserts, and milk. Pancreatic cases were older and showed the lowest daily energy intake among the three types of digestive cancer.

We observed an inverse association between the total dairy products consumption and esophageal cancer risk (*p* = 0.042). Compared to the lowest consumption group (low tertile, T1), moderate consumption (T2) of total dairy products was associated with a 41% lower risk of esophageal cancer (relative risk ratio (RRR) = 0.59, 95% confidence interval (95%CI) (0.37–0.96; *p*-trend = 0.081)) (Table 3). No associations were observed between total dairy products consumption and stomach and pancreatic cancers.

The consumption of fermented dairy was associated with a lower risk of esophageal cancer and stomach cancer (Table 4). Compared to the lowest consumption group (T1), participants in the highest consumption group (T3) showed a 45% lower risk of esophageal cancer (RRR_T3 vs. T1_ = 0.55 (0.33–0.90; *p*-trend = 0.009)) and a 32% lower risk of stomach cancer (RRR_T3 vs. T1_ = 0.68 (0.47–0.97; *p*-trend = 0.042)). Regarding pancreatic cancer, evidence of lower risk for fermented dairy consumption was observed, although the association was not significant (RRR_T3 vs. T1_ = 0.79 (0.49–1.28)).

Regarding the consumption of sugary dairy desserts, an 85% higher risk of stomach cancer was observed in the highest tertile (T3) of consumption compared with the lowest tertile (T1) (RRR _T3 vs. T1_ = 1.85 (95%CI: 1.30–2.64; *p*-trend ≤ 0.001)) (Table 4). No associations were found between total dairy products consumption and esophageal and pancreatic cancers. We did not find an association between the consumption of milk and the risk of any cancer (Table 4).

Despite the limited sample size of our study, we also explored the associations between dairy products and the main histological types of esophageal (i.e., squamous cell carcinoma and adenocarcinoma) and stomach cancers (i.e., intestinal and diffuse adenocarcinoma). In general, the estimates for histological types were similar to those observed for each cancer in Table 3 and Table 4, although the estimates were more unstable. We observed that the positive association with sugary dairy desserts was more evident for intestinal adenocarcinoma (RRR _T3 vs. T1_ = 2.24 (95%CI: 1.48–3.39)) (see Table S1).

## 4. Discussion

In this study, only moderate consumption of total dairy products was associated with a lower risk of esophageal cancer, while no association was observed for stomach and pancreatic cancers. However, the consumption of fermented dairy products (including yoghurt and cheese) was associated with a lower risk of both esophageal and stomach cancers. We also found that higher consumption of sugary dairy desserts was associated with a higher risk of stomach cancer. The consumption of milk was not associated with the risk of esophageal, stomach, or pancreatic cancers.

Few studies have examined the relationship between the consumption of total dairy products and esophageal cancer, and the results have been inconclusive. An overview of the reviews that included 52 studies, most of them meta-analyses or pooled analyses, suggested that higher consumption of total dairy products may be associated with decreased risk of gastrointestinal cancers, specifically colorectal cancer [26]. However, in contrast to our results, a recent analysis within the Prostate, Lung, Colorectal, and Ovarian Cancer Screening Trial, which included more than 100,000 individuals, showed no significant association between the consumption of total dairy products and esophageal cancer [27]. Similarly, a previous meta-analysis of observational studies found no significant association between the consumption of dairy products and esophageal squamous cell carcinoma [18]. These discrepancies among the studies described previously could be due to methodological aspects or differences in sample characteristics. While our study was a case–control study from Spain, other studies were conducted in the United States (USA) or Asia. Another reason could be that the total dairy food group does not always include the same components, and each dairy subgroup presents different characteristics and effects.

When we examined the association between subgroups of dairy products and the risk of esophageal, stomach, and pancreatic cancers, we observed that the consumption of fermented dairy, including yoghurt and cheese, was associated with a lower risk of esophageal and stomach cancers. A previous study, performed in three areas of the USA [28], explored the relationship between the consumption of different food groups (including both high- and low-fat dairy products) and esophageal and stomach cancers (including the following subtypes: esophageal adenocarcinoma and esophageal squamous cell carcinoma; gastric cardia adenocarcinoma and non-cardia gastric adenocarcinoma). While higher consumption of low-fat dairy products was associated with a lower risk of non-cardia gastric adenocarcinoma, greater consumption of high-fat dairy products was related to a higher risk of both types of esophageal cancer and gastric cardia adenocarcinoma [28]; however, in that study, the subtypes of dairy products were not differentiated beyond fat content, and therefore, the results may not be comparable with ours. A meta-analysis conducted by Zhang et al., which included 61 studies and almost 2 million participants [29], examined the relationship between fermented dairy products and overall cancer and subtypes. Although they did not find a statistically significant association between fermented dairy products and gastric cancer, greater consumption of fermented dairy products was associated with a lower risk of esophageal cancer (OR = 0.64, 95%CI: 0.54–0.77), which was in line with our findings. Other studies have also explored the association between yoghurt consumption and esophageal and stomach cancers, observing favorable results [30] or no associations [17], respectively.

Several mechanisms may explain the protective associations that we observed between total and fermented dairy products consumption and esophageal and stomach cancers in our study. Firstly, these food groups are rich sources of calcium and vitamin D, both of which have been linked to a reduced risk of gastrointestinal cancers [31,32,33]. It has been described how calcium might act by binding to bile acids and fatty acids in the gastrointestinal tract, thereby diminishing their potential cytotoxic effects on the mucosal lining [34]. Meanwhile, vitamin D appears to exhibit anti-proliferative effects on cancer cells and enhance immune function [35]. Secondly, dairy products contain conjugated linoleic acid and sphingolipids, which are compounds that have been shown to possess anti-carcinogenic properties in experimental studies [36,37,38]. Fermented dairy products in particular exert additional protective effects. Their probiotic content can modulate the gut microbiota and immune response, potentially influencing carcinogenesis in the gastrointestinal tract [39,40]. Probiotics such as *Lactobacillus* and *Bifidobacterium* species produce metabolites with anti-inflammatory and anti-carcinogenic properties, which contribute to maintaining the integrity of the epithelial barrier and to inhibiting the colonization of pathogenic bacteria [41,42,43]. In this sense, foods like fermented dairy products could also promote the formation of short-chain fatty acids, such as butyrate, which have been shown to inhibit cancer cell growth and induce apoptosis in cancerous cells, thereby reducing the risk of both esophageal and gastric cancers [44,45].

We also found that higher intake of sugary dairy desserts was associated with a greater risk of stomach cancer, particularly for the intestinal adenocarcinoma type. This subgroup of dairy products includes foods such as condensed milk, flan, and ice cream, which are considered ultra-processed foods according to the NOVA classification [46]. Previous studies using this kind of food have shown results similar to ours. In a hospital-based multicentric case–control study conducted in Brazil [47], the consumption of processed and ultra-processed foods, such as ice cream, was found to increase the risk of stomach cancer, particularly adenocarcinoma, as in our study. Similarly, a recent systematic review and meta-analysis [48] including more than 1 million participants showed a direct association between the highest consumption of ultra-processed foods and increased risk of colorectal and non-cardia stomach cancers.

Ultra-processed foods are low in nutritional quality and high in harmful substances like added sugars, unhealthy fats, and additives [46]. Their typically high calorie density can contribute to obesity, a known risk factor for stomach cancer [49]. Specifically, sugary dairy desserts may disrupt the gut microbiota, leading to dysbiosis and increased intestinal permeability, allowing toxins to enter the bloodstream and cause systemic inflammation, directly impacting the gastric mucosa and raising stomach cancer risk [50,51].

The consumption of milk was not associated with any cancer in our study. Other studies that explored this association have found inconsistent results. A meta-analysis exploring the association between milk consumption and gastric cancer risk among European populations demonstrated that higher milk consumption was not associated with gastric cancer, similar to the results of our study [19]. Our results also align with those of a pooled analysis of 14 prospective cohorts, in which no association between milk consumption and pancreatic cancer was found [52]. In contrast, the consumption of more than three servings of milk per week was associated with an increased risk of esophageal cancer death in a Chinese cohort study [53].

This study may have limitations. The sample size was limited, particularly for esophageal and pancreatic cancers, which may have influenced and reduced the statistical power to detect some associations. Case–control studies are also subject to biases, such as selection bias, although in our case, the participation rate was similarly high in cases and controls. Another common bias in case–control studies is misclassification bias, although the use of validated questionnaires would reduce its possible influence. Moreover, our results should be taken with caution, since the data in our study were collected 25 years ago. While the number and variety of dairy products may have changed during the elapsed period, we believe that, in essence, the main composition of dairy products, and particularly of the three subgroups considered in our study, should not be very different at present. Consequently, the lower risk of esophageal and stomach cancers found in the intake of fermented dairy products and the higher risk of stomach cancer observed in the intake of sugary dairy desserts could be real. However, these findings should be confirmed by further studies. Another limitation might concern the measurement of diet, as it refers to dietary intake from five years prior to diagnosis. We considered this period justified due to the likely appearance of symptoms before cancer diagnosis, which could lead patients to make changes in their diet. Thus, the 5-year reproducibility and validity of the FFQ was explored in a group of 80 adults, showing acceptable reproducibility (correlation coefficient average, r = 0.40) and validity using the mean of four one-week dietary records as the reference method (average r = 0.37) [23]. Lastly, although multivariable analyses accounted for various confounding factors, there may be other uncontrolled factors influencing the development of esophageal, stomach, and pancreatic cancers.

Our study has several strengths. Firstly, the fact that the observed associations persisted even after considering various confounding variables and established exposure factors, such as tobacco and alcohol consumption, highlights their robustness. Secondly, the use of a structured and validated FFQ [23] and the same protocol showing different associations for esophageal, stomach, and pancreatic cancers may reinforce our findings. Thirdly, we produced results consistent with other studies, adding new evidence for specific dairy products in a controversial area of research [10]. Finally, we also explored the association between dairy consumption and the histological subtypes of esophageal and stomach cancers, observing results consistent with the overall findings but more pronounced for certain subtypes.

## 5. Conclusions

In conclusion, this study suggests that higher consumption of fermented dairy products is associated with a lower risk of esophageal and stomach cancers, while higher consumption of sugary dairy desserts, such as custards or ice creams, is associated with a higher risk of stomach cancer. Further research is needed to confirm our results and accumulate more evidence, particularly regarding the effect of specific dairy products on every cancer.

## Figures and Tables

**Table 1:** Food items included in each dairy food group.

Food Group	Food Items Included
Total dairy products	Whole-fat milk, skimmed milk, condensed milk, yoghurt, cottage cheese, curd, white or fresh cheese, creamy cheese or cheese in portions, mature or semi-mature cheese (Manchego), custard, flan, pudding, ice cream
Dairy Subgroups	
1. Fermented dairy	Yoghurt, cottage cheese, curd, white or fresh cheese, creamy cheese or cheese in portions, mature or semi-mature cheese (Manchego)
2. Sugary dairy desserts	Condensed milk, custard, flan, pudding, ice cream
3. Milk	Whole-fat milk, skimmed milk

**Table 2:** Sociodemographic characteristics, lifestyle, and dairy products intake among controls and cases (esophagus, stomach, and pancreas) in the PANESOES study (*n* = 1229).

N.° of Participants (%)	Controls 455 (37.0)	Cases 774 (63.0)		
		Esophagus 199 (25.7)	Stomach 411 (53.1)	Pancreas 164 (21.2)
Age (years)	**63.0 (10.7)** ^1^	**60.5 (9.8)**	**64.8 (11.4)**	**65.4 (11.2)**
Sex, female (%)	**37.4**	**7.5**	**34.8**	**39.0**
Province, Valencia (%)	**69.5**	**77.4**	**68.1**	**64.0**
Educational level, <primary (%)	54.1	56.3	60.3	54.3
Alcohol, >99 g/d (%)	**3.1**	**26.6**	**4.4**	**6.1**
Energy intake (kcal/day)	**1861.9 (655.4)**	**2349.4 (851.2)**	**2067.0 (695.9)**	**2008.8 (771.6)**
Tobacco smoking, >24 c/d (%)	**7.2**	**32.2**	**10.2**	**13.4**
Fruit and vegetable intake (g/d)	**256.7 (106.6)**	**169.6 (96.1)**	**212.1 (97.7)**	**225.7 (103.3)**
Dairy products intake				
Total dairy products (g/d)	**322.4 (239.5)**	**266.7 (264.6)**	**367.9 (367.9)**	**339.7 (339.7)**
Fermented dairy products (g/d)	**39.6 (52.9)**	**26.4 (34.4)**	**36.1 (54.6)**	**32.1 (39.5)**
Sugary dairy desserts (g/d)	**9.8 (14.9)**	**10.3 (17.5)**	**14.3 (24.4)**	**11.9 (32.5)**
Milk (g/d)	**270.9 (217.3)**	**229.1 (246.7)**	**315.8 (261.4)**	**291.8 (232.0)**

Abbreviations: SD: standard deviation; g/d: grams/day; c/d: cigarettes/day. ^1^ Mean (SD) (all such values). Bold values represent *p*-value < 0.05.

**Table 3:** Association between the intake of total dairy products and cancers of the esophagus, stomach, and pancreas in participants of the PANESOES study (*n* = 1229).

	Total Dairy Products Intake (In Tertiles, g/day)				
	T1, <206 g/d	T2, 206–445 g/d	T3, >445 g/d		
		RRR (95%CI)	RRR (95%CI)	*p*-value ^2^	*p*-trend ^3^
Esophagus, *n* (cases/controls)	101/131	49/186	49/138		
Age, sex, and province adjusted	1.00	0.41 (0.27, 0.63)	0.52 (0.33, 0.80)		
Multivariable ^1^	1.00	0.59 (0.37, 0.96)	0.72 (0.43, 1.18)	0.042	0.081
Stomach, *n* (cases/controls)	125/131	126/186	160/138		
Age, sex, and province adjusted	1.00	0.74 (0.52, 1.04)	1.29 (0.91, 1.83)		
Multivariable ^1^	1.00	0.82 (0.57, 1.18)	1.21 (0.83, 1.75)	0.185	0.357
Pancreas, *n* (cases/controls)	53/131	49/186	62/138		
Age, sex, and province adjusted	1.00	0.66 (0.42, 1.05)	1.19 (0.75, 1.89)		
Multivariable ^1^	1.00	0.75 (0.47, 1.20)	1.22 (0.75, 1.99)	0.126	0.330

Abbreviations: T: tertile; RRR: relative risk ratio from multinomial logistic regression; 95%CI: 95% confidence intervals. ^1^ Multivariable model adjusted for age (<60; 60–69; >70 years), sex (male or female), province (Alicante and Valencia), educational level (<primary; primary; >primary), smoking (never smoked; ex-smoker; ≤24 c/day; ≥25 c/day), alcohol consumption (never; 1–24 g/d; 25–49 g/d; 50–99 g/d; >99 g/d), daily energy intake (kcals/d), and daily fruit and vegetable consumption (g/d). ^2^ *p*-value from the likelihood ratio test. ^3^ *p*-value from the trend test.

**Table 4:** Association between the intake of specific dairy products and cancers of the esophagus, stomach, and pancreas in participants of the PANESOES study (*n* = 1229).

	Specific Dairy Products Intake in Tertiles				
		RRR (95%CI)	RRR (95%CI)	*p*-value ^2^	*p*-trend ^3^
Fermented Dairy Products Intake (In Tertiles, g/day)					
	T1, <11 g/d	T2, 11–30 g/d	T3, >30 g/d		
Esophagus, *n* (cases/controls)	86/130	60/152	53/173		
Age, sex, and province adjusted	1.00	0.58 (0.38, 0.89)	0.45 (0.29, 0.69)		
Multivariable ^1^	1.00	0.64 (0.40, 1.03)	0.55 (0.33, 0.90)	0.032	0.009
Stomach, *n* (cases/controls)	141/130	143/152	127/173		
Age, sex, and province adjusted	1.00	0.90 (0.64, 1.26)	0.72 (0.52, 1.02)		
Multivariable ^1^	1.00	0.82 (0.58, 1.16)	0.68 (0.47, 0.97)	0.122	0.042
Pancreas, *n* (cases/controls)	54/130	54/152	56/173		
Age, sex, and province adjusted	1.00	0.90 (0.57, 1.41)	0.86 (0.55, 1.35)		
Multivariable ^1^	1.00	0.86 (0.54, 1.37)	0.79 (0.49, 1.28)	0.652	0.405
Sugary Dairy Desserts Intake (In Tertiles, g/day)					
	T1, <1.5 g/d	T2, 1.5–11.3 g/d	T3, >11.3 g/d		
Esophagus, *n* (cases/controls)	89/148	57/174	53/133		
Age, sex, and province adjusted	1.00	0.62 (0.41, 0.93)	0.75 (0.49, 1.15)		
Multivariable ^1^	1.00	0.82 (0.51, 1.32)	1.30 (0.80, 2.11)	0.479	0.521
Stomach, *n* (cases/controls)	119/148	127/174	165/133		
Age, sex, and province adjusted	1.00	0.93 (0.67, 1.31)	1.59 (1.13, 2.23)		
Multivariable ^1^	1.00	1.04 (0.73, 1.48)	1.85 (1.30, 2.64)	<0.001	<0.001
Pancreas, *n* (cases/controls)	54/148	51/174	59/133		
Age, sex, and province adjusted	1.00	0.81 (0.52, 1.27)	1.24 (0.79, 1.93)		
Multivariable ^1^	1.00	0.93 (0.59, 1.47)	1.45 (0.91, 2.30)	0.092	0.125
Milk Intake (In Tertiles, g/day)					
	T1, <170 g/d	T2, 170–409 g/d	T3, >409 g/d		
Esophagus, *n* (cases/controls)	100/137	48/173	51/145		
Age, sex, and province adjusted	1.00	0.48 (0.31, 0.73)	0.55 (0.36, 0.85)		
Multivariable ^1^	1.00	0.72 (0.45, 1.17)	0.74 (0.45, 1.22)	0.304	0.154
Stomach, *n* (cases/controls)	124/137	133/173	154/145		
Age, sex, and province adjusted	1.00	0.87 (0.62, 1.23)	1.23 (0.87, 1.73)		
Multivariable ^1^	1.00	0.98 (0.69, 1.40)	1.12 (0.77, 1.63)	0.839	0.588
Pancreas, *n* (cases/controls)	49/137	56/173	59/145		
Age, sex, and province adjusted	1.00	0.91 (0.58, 1.44)	1.20 (0.75, 1.92)		
Multivariable ^1^	1.00	1.07 (0.67, 1.72)	1.23 (0.75, 2.01)	0.708	0.407

Abbreviations: T: tertile; RRR: relative risk ratio from multinomial logistic regression; 95%CI: 95% confidence intervals. ^1^ Multivariable model adjusted for age (<60; 60–69; >70 years), sex (male or female), province (Alicante and Valencia), educational level (<primary; primary; >primary), smoking (never smoked; ex-smoker; ≤24 c/day; ≥25 c/day), alcohol consumption (never; 1–24 g/d; 25–49 g/d; 50–99 g/d; >99 g/d), daily energy intake (kcals/d), and daily fruit and vegetable consumption (g/d). ^2^ *p*-value from the likelihood ratio test. ^3^ *p*-value from the trend test.

## Data Availability

The data presented in this study are available on request from the corresponding author. The data are not publicly available due to confidentiality and ethical reasons.

## Supplementary Materials

The following supporting information can be downloaded at: https://www.mdpi.com/article/10.3390/cancers16244151/s1, Table S1: Association between the intake of total and specific dairy products and cancers of the esophagus and stomach by histopathological type in participants of the PANESOES study.
