# Supplementary material for: Association Between Dairy Products Consumption and Esophageal, Stomach, and Pancreatic Cancers in the PANESOES Multi Case–Control Study
Source: Cancers (Basel). 2024 Dec 12;16(24):4151. doi: 10.3390/cancers16244151 (PMC11674531; doi:10.3390/cancers16244151)
Supplement: Supplementary file 1 [file cancers-16-04151-s001.zip › cancers-3324083-supplementary.pdf]

Table S1. Association between the intake of **total and specific dairy products** and cancers of the esophagus and stomach by histopathological type in participants of the PANESOES study.

	Dairy Products Intake In Tertiles				
		RRR (95%CI)	RRR (95%CI)	p-value[2]	p-trend[3]
**Total Dairy Products Intake (In Tertiles, g/day)**					
	T1, < 206 g/d	T2, 206–445 g/d	T3, > 445 g/d		
Esophageal squamous cell carcinoma	1.00	0.59 (0.35, 1.00)	0.59 (0.33, 1.06)	0.053	**0.040**
Esophageal adenocarcinoma	1.00	0.50 (0.20, 1.28)	0.86 (0.37, 1.99)	0.168	0.359
Stomach intestinal adenocarcinoma	1.00	0.89 (0.59, 1.35)	1.20 (0.77, 1.85)	0.347	0.350
Stomach diffuse adenocarcinoma	1.00	0.67 (0.38, 1.18)	1.13 (0.65, 1.94)	0.396	0.722
**Fermented Dairy Products Intake (In Tertiles, g/day)**					
	T1, <11 g/d	T2, 11–30 g/d	T3, >30 g/d		
Esophageal squamous cell carcinoma	1.00	0.65 (0.38, 1.11)	**0.54 (0.31, 0.94)**	**0.046**	**0.013**
Esophageal adenocarcinoma	1.00	0.58 (0.25, 1.34)	0.48 (0.21, 1.13)	0.077	**0.024**
Stomach intestinal adenocarcinoma	1.00	0.88 (0.59, 1.30)	**0.58 (0.38, 0.89)**	0.068	**0.023**
Stomach diffuse adenocarcinoma	1.00	0.73 (0.43, 1.24)	0.74 (0.44, 1.26)	0.567	0.404
**Sugary Dairy Desserts Intake (In Tertiles, g/day)**					
	T1, <1.5 g/d	T2, 1.5–11.3 g/d	T3, >11.3 g/d		
Esophageal squamous cell carcinoma	1.00	0.84 (0.50, 1.43)	1.33 (0.77, 2.30)	0.585	0.654
Esophageal adenocarcinoma	1.00	0.90 (0.40, 2.05)	1.29 (0.58, 2.92)	0.418	0.345
Stomach intestinal adenocarcinoma	1.00	1.13 (0.75, 1.72)	**2.24 (1.48, 3.39)**	**<0.001**	**<0.001**
Stomach diffuse adenocarcinoma	1.00	1.24 (0.73, 2.08)	1.53 (0.89, 2.63)	0.187	0.067
**Milk Intake (In Tertiles, g/day)**					
	T1, <170 g/d	T2, 170–409 g/d	T3, >409 g/d		
Esophageal squamous cell carcinoma	1.00	0.72 (0.42, 1.23)	0.61 (0.34, 1.07)	0.241	0.094
Esophageal adenocarcinoma	1.00	0.60 (0.23, 1.53)	1.01 (0.44, 2.33)	0.360	0.497
Stomach intestinal adenocarcinoma	1.00	1.02 (0.67, 1.55)	1.17 (0.76, 1.80)	0.698	0.410
Stomach diffuse adenocarcinoma	1.00	0.83 (0.47, 1.44)	1.00 (0.58, 1.73)	0.926	0.875

Abbreviations: O: esophageal; S: stomach; T: tertile; RRR: *relative risk ratio* from multinomial logistic regression; 95%CI: 95% confidence intervals. Models adjusted for age (<60; 60–69; >70 years), sex (male or female), province (Alicante and Valencia), educational level (<primary; primary; >primary), smoking (never smoked; ex-smoker; ≤24c/day; ≥25c/day), alcohol consumption (never; 1–24 g/d; 25–49 g/d; 50–99 g/d; >99 g/d), daily energy intake (kcals/d), and daily fruit and vegetable consumption (g/d). [2] p-value from the likelihood ratio test. [3] p-value from the trend test. Number of participants by histopathological type: 157 esophageal squamous cell carcinoma; 42 esophageal adenocarcinoma; 242 stomach intestinal adenocarcinoma; 119 stomach diffuse adenocarcinoma.
